# Outcomes for children and young people growing up in kinship care in Scotland: A population-level data linkage study

**DOI:** 10.23889/ijpds.v8i2.2208

**Published:** 2023-09-14

**Authors:** Joanna Soraghan, Robert Porter

**Affiliations:** 1 CELCIS, University of Strathclyde, Glasgow, United Kingdom

## Objectives

The number of children and young people living in kinship care (living separately from their parents with a family friend/relative) in Scotland has increased consistently since 2010. This study aims to explore outcomes for children and young people in kinship care in terms of their health, education, and care journeys.

## Method

This population-wide data linkage study will utilise data on all children and young people who have been placed in formal kinship care from 2008 onwards. The datasets linked for this study are:

- Looked After Children
- Child Protection
- Health Visiting
- Educational attainment, attendances, absences and exclusion
- Children’s Hearings data

Through descriptive analysis the study will provide insight into the experiences and outcomes of children and young people in kinship care. Where appropriate, comparisons will be drawn with the wider care population and the general population of children and young people in Scotland.

## Results

Data will be presented which highlight:

- Levels of kinship care usage in Scotland, and how this varies regionally and over time.
- The routes into kinship care in terms of child protection concerns and legal reasons data.
- How children fare while in kinship care – specifically in terms of their childhood development, placement stability and educational attainment/engagement.The pathways out of formal kinship care, and whether these differ over time.
- The pathways out of formal kinship care, and whether these differ over time.

Full results will be available for publication at the 2023 ADR UK conference.

## Conclusion

This is the first population-wide study on outcomes for children and young people living in kinship care within Scotland. It is hoped that insight arising from this work will aid local authorities, government and others in meeting the needs of the steadily increasing number of children living in formal kinship care.

